# Acute unstable depressive syndrome (AUDS) is associated more frequently with epilepsy than major depression

**DOI:** 10.1186/1471-2377-10-67

**Published:** 2010-07-30

**Authors:** Arne E Vaaler, Gunnar Morken, Valentina C Iversen, Daniel Kondziella, Olav M Linaker

**Affiliations:** 1 Department of Neuroscience, Norwegian University of Science and Technology, Trondheim, Norway; 2 Department of Neurology, Rigshospitalet, Copenhagen University Hospital, Copenhagen, Denmark; 3 Division of Psychiatry, Haukeland University Hospital, Bergen, Norway; 4 Division of Psychiatry, Department Østmarka, St Olav's University Hospital, Trondheim, Norway; 5 Division of Psychiatry, Department of Research and Development, St Olav's University Hospital, Trondheim, Norway

## Abstract

**Background:**

Depressive disorders are frequent in epilepsy and associated with reduced seizure control. Almost 50% of interictal depressive disorders have to be classified as atypical depressions according to DSM-4 criteria. Research has mainly focused on depressive symptoms in defined populations with epilepsy (e.g., patients admitted to tertiary epilepsy centers). We have chosen the opposite approach. We hypothesized that it is possible to define by clinical means a subgroup of psychiatric patients with higher than expected prevalence of epilepsy and seizures. We hypothesized further that these patients present with an Acute Unstable Depressive Syndrome (AUDS) that does not meet DSM-IV criteria of a Major Depressive Episode (MDE). In a previous publication we have documented that AUDS patients indeed have more often a history of epileptic seizures and abnormal EEG recordings than MDE patients (Vaaler et al. 2009). This study aimed to further classify the differences of depressive symptoms at admittance and follow-up of patients with AUDS and MDE.

**Methods:**

16 AUDS patients and 16 age- and sex-matched MDE patients were assessed using the Symptomatic Organic Mental Disorder Assessment Scale (SOMAS), the Montgomery and Åsberg Depression Rating Scale (MADRS), and the Mini-Mental State Test (MMST), at day 2, day 4-6, day 14-16 and 3 months after admittance to a psychiatric emergency unit. Life events were assessed with The Social Readjustment Rating Scale (SRRS) and The Life Experience Survey (LES). We also screened for medication serum levels and illicit drug metabolites in urine.

**Results:**

AUDS patients had significantly higher SOMAS scores (average score at admission 6.6 ± 0.8), reflecting increased symptom fluctuation and motor agitation, and decreased insight and concern compared to MDE patients (2.9 ± 0.7; p < 0.001). Degree of mood depression, cognition, life events, drug abuse and medication did not differ between the two groups.

**Conclusions:**

AUDS patients present with rapidly fluctuating mood symptoms, motor agitation and relative lack of insight and concern. Seizures, epilepsy and EEG abnormalities are overrepresented in AUDS patients compared to MDE patients. We suggest that the study of AUDS patients may offer a new approach to better understanding epilepsy and its association with depressive disorders.

**Trial registration:**

NCT00201474

## Background

Depression is more frequent in patients with epilepsy compared to the general population [1,2]. Every second patient admitted to a tertiary epilepsy center may suffer from concurrent depressive disorders [3,4]. Depression has greater negative impact on quality of life than seizure frequency or the number of prescribed antiepileptic drugs [4-6]. In addition, depression in epilepsy is associated with reduced seizure control [7-10] and dramatically increased suicide risk [11-14] and health care costs [15]. Recent evidence suggests that the relationship between epilepsy and depression is bidirectional [2,16-18]. Thus, patients with epilepsy are more likely to develop depressive disorders, and patients with a history of depression or suicide attempt have a higher risk of developing epilepsy.

Interictal depressive disorders in patients with epilepsy have unique manifestations that are poorly reflected in the established classification systems ICD-10 and DSM-IV [19-24]. Symptoms such as suicidal ideation, frustration intolerance, irritability and motor agitation can rapidly alternate with symptom-free periods lasting one to several days. Blumer and co-workers have called this type of depression "an interictal dysphoric disorder with a characteristic intermittent and pleomorphic symptomatology" [23]. Kanner has termed it the "dysthymic-like disorder of epilepsy" [3]. Indeed, almost 50% of interictal depressive disorders may have to be classified as atypical depressions according to DSM-IV criteria [23]. It can be concluded that the physician must be cautious not to overlook atypical interictal mood disturbances. However, this sometimes leads to a well-known clinical dilemma in which the patient tends to attribute all mood disturbances to sub-clinical epileptic activity (and consequently demands revision of antiepileptic therapy), whereas the neurologist may not at all be confident with the patient's interpretation. Depression and many types of epilepsy share indeed several pathogenic mechanisms [7,8]. To determine when interictal mood disturbance in a given patient is due to sub-clinical epileptic activity and when not, is often impossible.

Clinical research on the association of depression and epilepsy has for the sake of simplicity focused on depressive symptoms in defined populations with epilepsy (e.g., patients admitted to a tertiary epilepsy center). This approach has the obvious disadvantage that the study population is highly pre-selected. In order to examine epilepsy and depression from a different angle, we have therefore chosen the opposite approach in the present study. We hypothesized that it is possible to define by clinical means a subgroup of psychiatric patients with a higher than expected prevalence of epilepsy and seizures. We hypothesized further that this patient group presents to the psychiatric emergency unit with rapidly fluctuating mood symptoms that do not meet DSM-IV criteria of a Major Depressive Episode (MDE). For the purpose of the present study we have called this presentation the Acute Unstable Depressive Syndrome (AUDS). In a prospective study we have been able to show that AUDS patients indeed more often have a history of epileptic seizures [25] and abnormal quantitative EEG recordings [26] than age- and sex-matched MDE patients. The main aim of the present paper was to further classify the differences of depressive symptoms at admittance and follow-up of patients with AUDS and MDE. For this purpose we have developed a 5-item diagnostic scale to measure the prevalence and degree of atypical depressive mood symptoms. We have called this scale the Symptomatic Organic Mental disorder Assessment Scale (SOMAS).

## Methods

### Study groups

During three years all patients (n = 1038) admitted to the psychiatric emergency unit (n = 1984) at the psychiatric division of St. Olav's University Hospital in Trondheim, Norway, were evaluated for inclusion in the present study. The following criteria had to be fulfilled:

A. AUDS group: Patients had to present with a rapidly developing psychiatric disorder starting within the previous 14 days. During this period the patient had to exhibit at some time symptoms that - with the important exception of the time criterion - met criteria for at least two diagnoses in the Diagnostic and Statistical Manual of Mental Disorders, fourth edition (DSM-IV) Axis 1 categories [20]. One of these DSM-IV Axis 1 categories had to be a depressive episode as defined by the Montgomery and Åsberg Depression Rating Scale (MADRS) ≥ 20 [27]. All patients were assessed in co-operation between experienced senior psychiatrists (AEV, GM, OML). One of the following criteria lead to exclusion: a psychiatric disorder due to intoxication; dementia or cognitive impairment to such a degree that informed consent could not be obtained; a diagnosis of unstable personality disorder with identical symptoms at former admissions; and lack of sufficient knowledge of the English or Norwegian language. 28 (2.7%) of the patients fulfilled the criteria for inclusion in the AUDS group. Twelve of these were excluded due to the reasons stated above. Sixteen AUDS patients entered the study. The co-diagnoses (with exception of the time criterion) in this group were DSM-IV 298.8 "brief psychotic disorder" (9 patients), DSM-IV 300.1 "panic disorder" (4 patients) and DSM-IV 296.0 "single manic episode" (3 patients).

B. MDE group: This group consisted of acutely admitted sex- and age-matched (+/- 5 years) patients meeting criteria for current Axis 1 major depressive episode (MADRS ≥20). After inclusion of a patient in the AUDS group, the first patient meeting MDE criteria and giving informed consent to participate in the study was recruited to the MDE group (n = 16).

### Group assessments

Our pre-study clinical observations had indicated that characteristic features of AUDS included fluctuating psychiatric symptoms, suicidal ideation, motor agitation (or relative lack of psychomotor retardation) when depressed, and lack of ability to understand or explain present symptoms. To test the validity of our observations we developed a 5-item scale with operational scoring criteria for estimating prevalence and degree of the symptoms we had noted. This is the Symptomatic Organic Mental disorder Assessment Scale (SOMAS). In a pre-study of patients consecutively admitted to the psychiatric emergency unit (n = 22), SOMAS showed good inter-rater reliability and internal consistency. It contains two main factors, one related to motor symptoms (motor retardation versus agitation), and one related to the insight and interest in one's own disorder (considerable effort, or lack thereof, in finding an explanation). Compared to MADRS, SOMAS measures a different concept than depression. Item A is measuring the degree of observable fluctuation in symptoms during the previous 24 hours. Item B is measuring the degree of motor retardation and item C the degree of increased motor activity during the last 24 hours. Items B and C, assessed at the time the patient is considered most depressed, are modified from The Positive and Negative Syndrome Scale (PANSS) for schizophrenia [28]. Item D is measuring the degree of insight the patient has about his/her affective symptoms. Item E is measuring the effort the patient makes in finding an explanation for these symptoms. Further information about Items A-E and the scoring procedure can be found in Appendix.

Patients were assessed using SOMAS, MADRS [27] and the Mini-Mental State Test (MMST) [29] at four different time points: day 2, day 4-6, day 14-16 and 3 months after admittance. A MMST score < 25 was regarded as a clinically relevant cognitive reduction [30]. Life events during the 6 months prior to admission were rated with The Social Readjustment Rating Scale (SRRS) [31], and life changes during the 12 months prior to admittance with a 24-item version of The Life Experience Survey (LES) [32]. These scales allow for detection of positive and negative life experiences as well as their impact on the patient. Alcohol consumption was assessed with The Alcohol Use Identification Test (AUDIT) [33]. Current medication concentrations were analyzed in blood samples, and drug use was screened for by urine samples at admittance. During admissions the patients were examined using MRI and serial EEG-videometry as described previously [25,26]. Diagnoses were set according to The Structural Clinical Interview for DSM-IV (SCID-1) [34].

### Statistical methods

Statistical differences between groups were analyzed using the Student's t-test and the Fisher exact test with two-tailed p < 0.05 considered significant.

### Ethics

All patients gave written informed consent. The study was conducted in accordance with the Declaration of Helsinki and approved by The Regional Medical Research Ethics Committee, Central Norway.

## Results

The clinical background data have been published previously [25]. The data are summarized in Table 1. AUDS patients had significantly more often a history of seizures and a diagnosis of epilepsy. They also had significantly more often epileptiform activity and focal or generalized slow activity on standard EEG.

**Table 1:** Clinical background data of AUDS and MDE patients

	AUDS patients (n = 16; age 32y ± 11; 6 males)	MDE patients (n = 16; age 33y ± 13; 6 males)	P value*
Problematic alcohol consumption	4 (n = 15)	4 (n = 13)	ns
Alcohol withdrawal symptoms	0	3	ns
Drug abuse	1^&^	0	ns
Benzodiazepine medication	6	3	ns
Anti-epileptic medication for epilepsy ^#^	4	0	ns
Anti-epileptic medication for other indications ^#^	3	0	ns
Seizures in clinical history	6	0	0.018
Fulfilling clinical criteria for epilepsy	5	0	0.043
Focal or generalized slow, or epileptiform EEG activity (≥ 2 pathological features)	8	1	0.015
Cerebral MRI pathology	2 (n = 13)	2 (n = 13)	ns
Pathological findings at neurological bedside examination indicative of CNS pathology	3 (n = 11)	1 (n = 13)	ns

The depicted data have been published elsewhere by our group. For further information on the data and the methods, see *Vaaler AE et al. BMC Psychiatry. 2009; 9: 63 *[25]. *Fisher exact tests. ^# ^Of a total of 7 patients, 3 were on lamotrigine, 3 on carbamazepine, 1 was on valproate. ^&^Cannabis. AUDS: Acute Unstable Depressive Syndrome; MDE: Major Depressive Episode; ns: not significant.

In the five AUDS patients fulfilling clinical criteria for epilepsy, two had juvenile generalised epilepsy syndromes, both with generalized tonic-clonic (GTC) seizures and absences. One had a syndrome of posttraumatic localisation-related epilepsy with nocturnal seizures presumed to be secondarily generalized, and two had scattered GTCs with a syndromic classification that remained undetermined [25].

At clinical neurological examination, three AUDS patients and one control patient had signs of CNS pathology. One AUDS patient had ophtalmoplegia and bilaterally inverted plantar reflexes (positive Babinski's sign), whereas another had inverted plantar reflexes as the only sign of pathology. The third patient had bilateral horizontal nystagmus. In the control group, there was a unilaterally inverted plantar reflex in one patient [25].

The AUDS patients had significantly higher temporal delta band amplitude than the MDE patients. Delta asymmetry was larger in AUDS than MDE in all regions. The delta activity dominated in the right hemisphere. Twelve of 16 AUDS patients had temporal delta amplitude above 8.9 μV as compared to 3 of 16 MDE patients. Three MDE - and 11 AUDS patients had absolute temporal delta amplitude side difference above 0.1 μV [26].

There were no statistical differences between AUDS and MDE patients with respect to antiepileptic medication, benzodiazepine intake, drug abuse, alcohol consumption and alcohol withdrawal symptoms.

Results from SOMAS are shown in Table 2. At admission and during the first 2 weeks AUDS patients had significantly higher SOMAS scores for any given item (average score at admission 6.6 ± 0.8), reflecting increased symptom fluctuation and motor agitation, and decreased insight and concern compared to MDE patients (2.9 ± 0.7; p < 0.001). At three months follow-up AUDS patients did no longer exhibit symptom fluctuation. Yet they still scored high for motor restlessness and lack of concern, although on a lower level.

**Table 2:** AUDS and MDE groups assessed by the Symptomatic Organic Mental Disorder Assessment Scale (SOMAS)

SOMAS	2 days after Admittance			4-6 days after admittance			14-16 days after admittance			3 months after admittance		
	**AUDS**	**MDE**	**P value**	**AUDS**	**MDE**	**P value**	**AUDS**	**MDE**	**P value**	**AUDS**	**MDE**	**P value**

Item A	5.7 ± 1.5	2.3 ± 0.8	<0.001	4.6 ± 1.7	2.1 ± 0.7	<0.001	4.5 ± 2.0	1.8 ± 0.8	<0.001	2.3 ± 1.2	1.7 ± 0.6	ns
Item B	9.9 ± 0.5	6.3 ± 2.0	<0.001	9.6 ± 1.3	6.9 ± 1.7	<0.001	9.7 ± 1.3	7.2 ± 1.7	<0.001	9.7 ± 1.0	7.9 ± 2.0	0.007
Item C	5.8 ± 2.3	1.2 ± 0.5	<0.001	4.0 ± 2.2	1.1 ± 0.5	<0.001	4.4 ± 2.3	1.1 ± 0.3	<0.001	3.1 ± 1.5	1.2 ± 0.6	<0.001
Item D	7.8 ± 1.8	2.1 ± 1.1	<0.001	7.7 ± 1.5	2.0 ± 0.9	<0.001	7.6 ± 1.7	1.9 ± 0.9	<0.001	6.9 ± 1.8	2.3 ± 1.7	<0.001
Item E	3.6 ± 1.6	2.6 ± 1.2	0.04	4.0 ± 1.6	2.2 ± 1.1	0.002	3.9 ± 1.4	2.6 ± 1.1	0.008	4.7 ± 1.4	2.2 ± 0.8	<0.001

Average item A-E	6.6 ± 0.8	2.9 ± 0.7	<0.001	6.0 ± 1.0	2.9 ± 0.6	<0.001	6.0 ± 1.1	2.9 ± 0.6	<0.001	5.4 ± 0.8	3.1 ± 0.7	<0.001

AUDS and MDE groups were assessed using SOMAS at four different time points. For each item (A-E) a score from 0 to 10 was given. All values are shown as mean ± sd. For details consider *Methods *and *Appendix*. AUDS: Acute Unstable Depressive Syndrome; MDE: Major Depressive Episode; ns: not significant.

MADRS, which measures the degree of mood depression, did not reveal any significant differences (Table 3). Cognition, life events and life style changes (assessed as MMST < 25, SRRS and LES, respectively) did not differ between AUDS and MDE patients (data not shown).

**Table 3:** AUDS and MDE groups assessed by the Montgomery and Åsberg Depression Rating Scale (MADRS)

Time point after admittance	AUDS	MDE	P value
Day 2	29.2 ± 8.9	34.6 ± 7.1	ns
Day 4-6	19.5 ± 8.9	22.7 ± 4.6	ns
Day 14-16	17.2 ± 10.5	19.0 ± 8.6	ns
3 months	9.5 ± 7.0	10.6 ± 6.9	ns

AUDS and MDE groups were assessed using MADRS at four different time points. MADRS has a scoring scale that ranges from 0 (least depressed) to 60 (most depressed). A depressive episode is defined as MADRS ≥ 20. All values are shown as mean ± sd. For details consider *Methods*. AUDS: Acute Unstable Depressive Syndrome; MDE: Major Depressive Episode; ns: not significant.

## Discussion

We have compared two groups of patients with depressive disorders admitted to a psychiatric emergency unit. AUDS patients presented with rapidly fluctuating mood disturbances, motor agitation and relative lack of insight and concern, whereas MDE patients showed rather stable mood levels, motor retardation and better understanding of and greater interest in their symptoms. Differences between AUDS and MDE patients were present until remittance of the affective episodes. This may indicate that AUDS and MDE are clinically distinct syndromes. AUDS patients had significantly more often a history of seizures and diagnose of epilepsy as well as more abnormalities on standard and quantitative EEG. Hence, atypical depressive disorders are not only frequent in patients in tertiary epilepsy centers [35], but seizures, epilepsy and EEG abnormalities are also overrepresented in AUDS patients in psychiatric emergency units.

Our pre-study observations indicated that SOMAS measures a different concept than depression. This was supported by the present study. Whereas no differences between groups were observed with MADRS (suggesting that the level of mood decrease was the same), all SOMAS items were significantly different between AUDS and MDE patients at almost any given time point. Symptom fluctuation (Item A) was the only item that was no longer different between groups at 3 months follow-up. Motor agitation and lack of insight (Item B-D) gradually diminished during the observation period, but remained rather pronounced until the end. Agitation, defined as unusual motor restlessness accompanied by emotional tension, is frequently encountered in depressive disorders [36,37]. The present data indicate that in AUDS patients, agitation is a core symptom. The presence of agitation has clinical implications for the choice between antidepressants and antiepileptic mood stabilizers [36-39]. Antiepileptic mood stabilizers have a positive effect on agitated depression with or without organic brain disorders [36,40], but antidepressants are sometimes detrimental in these conditions [38,39]. Interestingly, whereas symptom fluctuation and motor agitation decreased in AUDS patients with time, lack of understanding (Item D) increased during the observation period. At 3 months follow-up AUDS patients often stated that they had given up searching for an explanation and did not want to put further concerns into it. This is perhaps in analogy to patients with epilepsy who more often than others believe that their life is controlled by external factors (fate, luck, chance) rather than internal factors (efforts, skills) [41,42]. It can be concluded that atypical depressive symptoms cannot be classified by DSM-IV and that SOMAS provides different information than MADRAS. The advantage of SOMAS is that it can be used to evaluate and classify clinical symptoms suggestive of AUDS.

The interictal dysphoric disorder has a characteristic chronic intermittent and pleomorphic symptomatology [23]. Also the AUDS clinical syndrome features pleomorphic psychiatric symptoms, but it differs from interictal dysphoric disorder in that it has a more acute onset and a more varied symptomatology including behaviorioral problems such as aggression. Therefore, patients with AUDS require acute psychiatric care. However, episodic interictal psychiatric conditions resembling interictal dysphoric disorder with slightly different characteristics are described and termed by different authors in the same manner as the AUDS [3,23,43]. Core characteristics of the AUDS clinical picture has similarities to some of the symptoms in these conditions, for instance, according to Himmelhoch such patients have "brief depressive dips with impulsive suicide attempts" [43].

Clinical observations made by neurologists, psychiatrists and epileptologists of epilepsy-specific psychiatric syndromes have led to an expert consensus proposing a new system of classification of these disorders [21]. These proposals are based on data primarily generated in tertiary epilepsy centers. The present study suggests that possibly, epilepsy-specific psychiatric syndromes might also be identified in psychiatric emergency units. Obviously, much larger patient populations must be studied before such a claim can be verified. However, our data indicate that assessment of AUDS patients is a possible approach to this issue in the future.

This study has a number of limitations and thus, the present results must be interpreted with caution. Patients were recruited in daily routine clinical practice and the ward staff was not blinded to the outcome of our assessment. We included patients with substance abuse, alcohol withdrawal symptoms, antiepileptic drug treatment and benzodiazepine intake. These factors probably affected the expression of symptoms as well as EEG recordings. However, in order to obtain a more naturalistic study, we chose not to exclude these patients [44]. The small number of subjects led to relatively weak statistical power. However, nearly all SOMAS-scores reached statistical significance, which indicates a strong correlation between epileptic activity, depression and the different SOMAS items. Further, one might argue that our study was affected by a pre-selection bias. Yet as stated earlier, previous studies on the prevalence of depressive symptoms in epilepsy populations have been affected by a similar selection bias.

The study also has some significant advantages. It was a prospective study in a naturalistic patient population from a defined catchments area. All patients admitted within a three-year period were evaluated for inclusion. We have used a robust validated instrument with documented sensitivity to symptom fluctuation [27]. All patients had EEG recordings shortly after admittance. We have extensively screened for alcohol and illicit drug use and medication serum levels. Moreover, we screened for a broad range of confounding factors. Cognition, life events and life style changes were assessed, but there were no differences between the groups.

## Conclusions

AUDS patients present with rapidly fluctuating mood symptoms, motor agitation when depressed and relative lack of insight and concern. These patients have more often a history of seizures, diagnose of epilepsy and EEG abnormalities than MDE patients. Whereas atypical depressive disorders are frequently encountered in patients in tertiary epilepsy centers [35], it seems that seizures, epilepsy and EEG abnormalities are also overrepresented in AUDS patients in psychiatric emergency units. Obviously, further studies are needed to corroborate these results. In a sentinel paper on epilepsy and depression, Kanner complains that "neurologists and psychiatrists [have] stopped talking to each other" [45]. It is time that both specialties join forces again. We suggest that the study of AUDS patients may offer a new approach to better understanding epilepsy and its association with depressive disorders.

## Competing interests

None of the authors has any financial interests or other potential conflicts of interests.

The study received funding from GlaxoSmithKline (GSK).

## Authors' contributions

AEV, GM, VCI and OML conceived, designed, and coordinated the study, examined and included the patients and helped to draft the manuscript. DK analyzed and interpreted the data and helped to draft the manuscript. All authors read and approved the final manuscript.

## Appendix

### Symptomatic Organic Mental Disorder Assessment Scale (SOMAS)

The ratings of all items are based on the investigator's personal examination of the patient, and/or information in the case records and/or information from the hospital ward staff.

The scorings are 1 - 10 on the following 5 items:

#### A: Degree of observable change in symptoms during the previous 24 hours

1: The symptoms have been completely stable throughout 24 hours.

3: Minor changes in symptoms during the past 24 hours (e.g., increased symptoms in the morning as in a depressive episode).

5: Some change of symptoms (e.g., breakthrough of depressive symptoms in hypomania).

8: Frequent alternation of symptoms, dominating more than half of the day.

10: Rapid fluctuation of symptoms from one half hour to the next.

#### B: Degree of motor retardation, rated during the period or periods of the previous 24 hours in which the patient was most depressed (modified from PANSS [28])

1: The patient has been almost completely immobile and virtually unresponsive to external stimuli.

3: Movements are extremely slow, resulting in a minimum of activity and speech. The patient is mostly sitting idly or lying down.

5: The patient has slow movements, and speech may be characterized by poor productivity, including long response latency, extended pauses, or slow pace.

8: Slight diminution in rate of movements and speech.

10: No motor retardation.

#### C: Degree of increased motor activity, rated during the period or periods the previous 24 hours when the patient was most depressed (modified from PANSS [28])

1: No increased motor activity.

3: The patient is slightly agitated with hypervigilance or has a tendency towards mild overarousal. The speech is slightly pressured.

5: The patient is clearly agitated and overaroused with affected speech and motor activity.

8: Marked excitement dominates the period and restricts attention and vital functions such as eating and sleeping.

10: The excitement is so extreme that interpersonal interaction is virtually impossible. The patient has acceleration of speech and motor activity resulting in incoherence and exhaustion.

#### D: Degree of patient's insight into his or her condition/symptoms

1: Mature and thoroughly considered attempt at explaining the condition. This explanation may or may not be psychotic.

3: The patient has been thinking of various possible explanations and has come up with a well-founded opinion about some of them.

5: The patient wonders about different causes of the condition, but is unsure.

8: The patient has one or several ideas about the cause, without any considered argumentation.

10: Patient is totally bewildered to what has happened or to what causes the condition.

X: Not possible to score; e.g. due to incapability to communicate verbally.

#### E: Degree of the patient's concern in finding an explanation for his or her condition/symptoms

1: Considerable engagement in finding an explanation of the condition.

3: Moderate engagement in finding an explanation of the condition.

5: Some engagement in finding an explanation of the condition.

8: Minimal engagement in finding an explanation of the condition.

10: The patient does not wonder at all what may have caused the condition.

X: Not possible to score; e.g. due to incapability to communicate verbally.

## Pre-publication history

The pre-publication history for this paper can be accessed here:

http://www.biomedcentral.com/1471-2377/10/67/prepub
